# Sirtuin 1 Inhibits Fatty Acid Synthesis through Forkhead Box Protein O1-Mediated Adipose Triglyceride Lipase Expression in Goat Mammary Epithelial Cells

**DOI:** 10.3390/ijms25189923

**Published:** 2024-09-14

**Authors:** Qiuya He, Weiwei Yao, Li Lv, Xuelin Zhang, Jiao Wu, Jun Luo

**Affiliations:** Shaanxi Key Laboratory of Molecular Biology for Agriculture, College of Animal Science and Technology, Northwest A&F University, Yangling, Xianyang 712100, China; heqiuya@nwafu.edu.cn (Q.H.); yaoweiwei@nwafu.edu.cn (W.Y.); lvli@nwafu.edu.cn (L.L.); 2444945765@nwafu.edu.cn (X.Z.); wujiao2019@nwafu.edu.cn (J.W.)

**Keywords:** SIRT1, dairy goat, transcriptional regulation, fatty acid synthesis, lactation

## Abstract

Sirtuin 1 (SIRT1) is a key upstream regulator of lipid metabolism; however, the molecular mechanisms by which SIRT1 regulates milk fat synthesis in dairy goats remain unclear. This study aimed to investigate the regulatory roles of SIRT1 in modulating lipid metabolism in goat mammary epithelial cells (GMECs) and its impact on the adipose triglyceride lipase (ATGL) promoter activity using RNA interference (RNAi) and gene overexpression techniques. The results showed that SIRT1 is significantly upregulated during lactation compared to the dry period. Additionally, SIRT1 knockdown notably increased the expressions of genes related to fatty acid synthesis (SREBP1, SCD1, FASN, ELOVL6), triacylglycerol (TAG) production (DGAT2, AGPAT6), and lipid droplet formation (PLIN2). Consistent with the transcriptional changes, SIRT1 knockdown significantly increased the intracellular contents of TAG and cholesterol and the lipid droplet abundance in the GMECs, while SIRT1 overexpression had the opposite effects. Furthermore, the co-overexpression of SIRT1 and Forkhead box protein O1 (FOXO1) led to a more pronounced increase in ATGL promoter activity, and the ability of SIRT1 to enhance ATGL promoter activity was nearly abolished when the FOXO1 binding sites (FKH1 and FKH2) were mutated, indicating that SIRT1 enhances the transcriptional activity of ATGL via the FKH element in the ATGL promoter. Collectively, our data reveal that SIRT1 enhances the transcriptional activity of ATGL through the FOXO1 binding sites located in the ATGL promoter, thereby regulating lipid metabolism. These findings provide novel insights into the role of SIRT1 in fatty acid metabolism in dairy goats.

## 1. Introduction

Goat milk and its products are favored by consumers for their high nutritional value, easy digestibility, and low allergy potential [1]. Our recent findings demonstrate that dietary intervention with goat milk can reduce blood glucose levels in type 2 diabetic mice and contribute to the restoration of the pancreatic function [2]. Additionally, fatty acid synthesis is a crucial metabolic process in the mammary gland that provides essential lipids required for milk production. The regulation of this process is complex, involving the coordinated actions of various signaling pathways and transcriptional regulators. Therefore, exploring the regulatory mechanism of lipid metabolism in the mammary glands of dairy goats is vital for improving the quality of goat milk.

Sirtuins1 (SIRT1) is a nicotinamide adenine dinucleotide (NAD+)-dependent protein deacetylase that plays crucial roles in regulating cellular metabolism, stress responses, and lipid metabolism [3,4,5]. In the mouse liver, SIRT1 has been reported to influence fatty acid synthesis [6]. Previous studies have suggested that SIRT1 can interact with and deacetylate the transcription factor *FOXO1*, which regulates the expressions of genes involved in lipid homeostasis [7,8,9]. Furthermore, SIRT1 reduces lipid accumulation by suppressing the expression of *SREBP1* in the liver [10,11] and activating *PGC-1α*, which enhances mitochondrial fatty acid oxidation [12]. These findings suggested that *SIRT1* is a key regulator of lipid metabolism. However, the underlying mechanisms by which SIRT1 regulates fatty acid synthesis in goat mammary epithelial cells (GMECs) remain largely unexplored.

Adipose triglyceride lipase (ATGL) is the rate-limiting enzyme in triglyceride hydrolysis that plays a key role in lipid droplet breakdown and triglyceride degradation, particularly in tissues with active lipogenesis [13]. The overexpression of *ATGL* in GMECs inhibits the fatty acid synthesis and lipid droplet accumulation [14], suggesting that ATGL plays an important role in fatty acid metabolism. Moreover, our recent study showed that *FOXO1* promotes *ATGL* promoter activity by binding to the FOXO1 binding sites of the *ATGL* promoters in GMECs [15]. Therefore, it is plausible that SIRT1 could influence fatty acid synthesis in GMECs through the FOXO1-mediated regulation of ATGL expression.

The main objectives of this study were to investigate the role of SIRT1 in the regulation of fatty acid synthesis in GMECs and to elucidate the underlying molecular mechanisms, with a focus on the SIRT1-FOXO1-ATGL axis.

## 2. Results

### 2.1. SIRT1 Expression across Different Tissues and Lactation Stages

**Figure 1:**
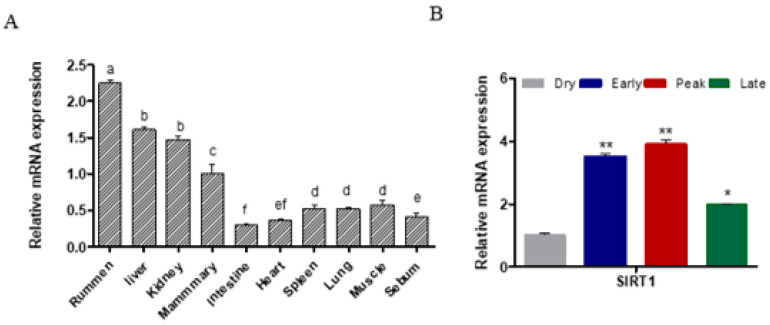
SIRT1 mRNA expression in goat tissues. (**A**) SIRT1 was expressed across multiple goat tissues. Lowercase letters indicate significant differences between groups (*p* < 0.05), while the same letters indicate no significant differences. (**B**) SIRT1 expression was significantly higher in the mammary gland during peak lactation compared to the dry period. The data were normalized to the dry period. The values are shown as the mean ± standard error of the mean (SEM) for three biological replicates. *, *p* < 0.05; **, *p* < 0.01.

*SIRT1* is broadly expressed across multiple tissues, with the highest levels observed in the rumen (*p* < 0.05; Figure 1A). Notably, substantial *SIRT1* expression was also detected in mammary gland tissue, which is crucial for milk production during lactation (Figure 1A). Interestingly, the *SIRT1* expression was dynamically regulated throughout the lactation cycle. During peak lactation, the *SIRT1* expression was significantly elevated compared to the dry period (*p* < 0.01; Figure 1B). These findings suggest that SIRT1 may play a vital regulatory role in adapting to the high metabolic demands of lactation in goats.

### 2.2. SIRT1 Deficiency Increases the Expressions of Genes Involved in Fatty Acid Synthesis, TAG Synthesis, and Lipid Droplet Formation

**Figure 2:**
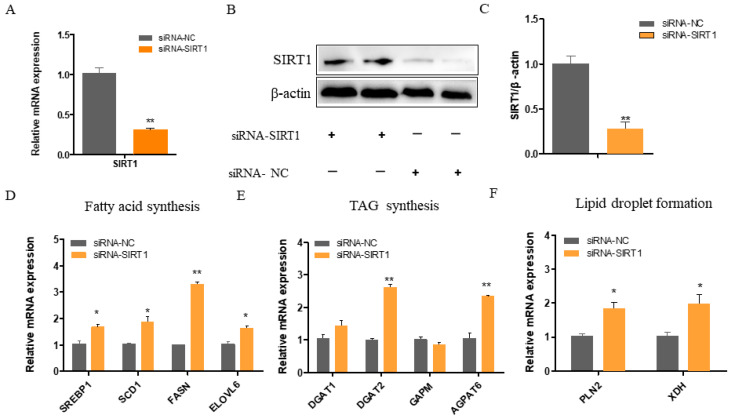
Effect of SIRT1 knockdown on the expressions of genes related to fatty acid metabolism in GMECs. (**A**) The mRNA levels of SIRT1 after infection with siRNA-SIRT1. (**B**,**C**) Protein level and quantification of SIRT1 in cells transfected with siRNA-SIRT1. The effect of SIRT1 knockdown on the expressions of genes involved in (**D**) fatty acid synthesis (SREBP1, SCD1, FASN, and ELOVL6), (**E**) TAG synthesis (DGAT1, DGAT2, GPAM, and AGPAT6), and (**F**) lipid droplet formation (PLIN2 and XDH). The RT-qPCR data were calculated using the 2^−ΔΔCt^ method. Data shown are mean ± SEM. Statistically significant differences are indicated: ** *p* < 0.01; * *p* < 0.05. Note: SIRT1, Sirtuin 1; GMECs, goat mammary epithelial cells; SREBP1, Sterol Regulatory Element-Binding Proteins 1; SCD1, stearoyl-coenzyme A desaturase; FASN, fatty acid synthase; ELOVL6, elongase of very long chain fatty acid 6; DGAT1/2, Diacylglycerol-O-Acyltransferase; GPAM, glycerol-3-phosphate acyltransferase; AGPAT6, 1-Acylglycerol-3-Phosphate O-Acyltransferase 6; PLIN2, Perilipin 2; XDH, xanthine dehydrogenase.

The siRNA-mediated knockdown resulted in a significant downregulation of the *SIRT1* mRNA levels, exceeding 60% compared to the control cells (*p* < 0.01; Figure 2A). Consistent with the diminished *SIRT1* mRNA levels, the SIRT1 protein expression was also markedly reduced in the knockdown group (*p* < 0.01; Figure 2B,C). Consequently, SIRT1 knockdown led to a significant increase in the mRNA expressions of *SREBP1*, *FASN* (*p* < 0.01), *SCD1*, and *ELOVL6* (*p* < 0.05; Figure 2D). Moreover, SIRT1 knockdown significantly increased the expressions of *DGAT2* and *AGPAT6* (*p* < 0.05), while it had no effect on the expression of *GPAM* (Figure 2E). Furthermore, the expressions of the lipid droplet formation-related genes *PLIN2* and *XDH* were significantly enhanced following *SIRT1* knockdown (*p* < 0.05; Figure 2F).

### 2.3. SIRT1 Overexpression Inhibits Genes Related to Fatty Acid Metabolism and Lipid Accumulation in GMECs

**Figure 3:**
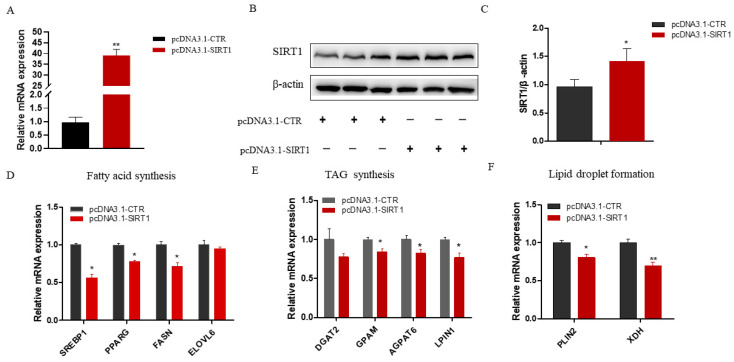
Effect of SIRT1 overexpression on the expressions of genes related to lipid metabolism in GMECs. (**A**) The SIRT1 mRNA expression in GMECs that were transfected with SIRT1 overexpression vector. (**B**,**C**) Protein abundance of SIRT1 after transfection of the pcDNA3.1-SIRT1 in GMECs (N = 3). The expressions of genes involved in lipid metabolism after SIRT1 overexpression including fatty acid synthesis (SREBP1, PPARG, FASN, and ELOVL6; panel (**D**)), TAG synthesis (DGAT2, GPAM, AGPAT6, and LIPIN1; panel (**E**)), and lipid droplet formation (PLIN2 and XDH; panel (**F**)). Data shown are mean ± SEM. Statistically significant differences are indicated: ** *p* < 0.01; * *p* < 0.05. Note: SIRT1, Sirtuin 1; SREBP1, Sterol Regulatory Element-Binding Proteins 1; PPARG, peroxisome proliferator-activated receptor gamma; FASN, fatty acid synthase; ELOVL6, elongase of very long chain fatty acid 6; DGAT2, Diacylglycerol-O-Acyltransferase 2; GPAM, glycerol-3-phosphate acyltransferase; AGPAT6, 1-Acylglycerol-3-Phosphate O-Acyltransferase 6; PLIN2, Perilipin 2; XDH, xanthine dehydrogenase.

To further investigate the functional roles of SIRT1 in GMECs, we constructed an overexpression vector and transfected it into the cells for 48 h. Compared with the control group, the SIRT1 expression was significantly elevated at both the mRNA (*p* < 0.01; Figure 3A) and protein (*p* < 0.01; Figure 3B,C) levels in the SIRT1 overexpression group. Subsequently, we examined the expressions of genes related to fatty acid synthesis. The results showed that the overexpression of *SIRT1* significantly downregulated the expressions of de novo fatty acid synthesis genes, including *SREBP1*, *FASN*, and *PPARG* (*p* < 0.05; Figure 3D). Additionally, the expressions of *GPAM*, *AGPAT6*, and *LIPIN1*, which are involved in triacylglycerol (TAG) synthesis, were also reduced (*p* < 0.05; Figure 3E). Moreover, the expressions of *PLIN2* and *XDH* were significantly decreased in the *SIRT1* overexpression group (*p* < 0.05; Figure 3F). These findings indicate that increased SIRT1 expression in GMECs can effectively suppress the expressions of genes critical for fatty acid synthesis and lipid droplet formation.

### 2.4. Manipulating SIRT1 Alters Intracellular TAG and Lipid Droplet Levels

**Figure 4:**
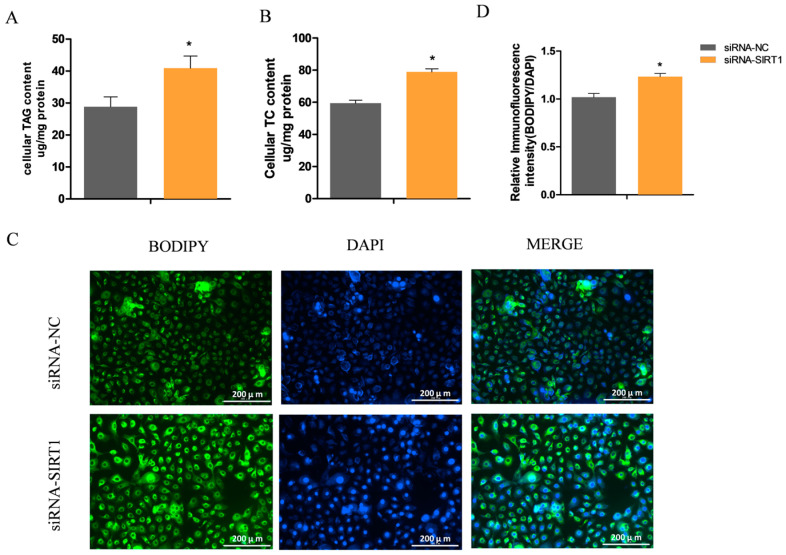
The effect of SIRT1 knockdown on the contents of triglycerides, cholesterol, and lipid droplets. (**A**) The content of triglycerides in cells transfected with siRNA-SIRT1. (**B**) Cellular cholesterol levels after knockdown of SIRT1 in GMECs. (**C**,**D**) Lipid droplets in SIRT1 knockdown cells were detected by BODIPY 493/503 staining. Scale bar = 200 μm.Values are shown as mean ± SEM. *, *p* < 0.05. Note: SIRT1, Sirtuin 1.

**Figure 5:**
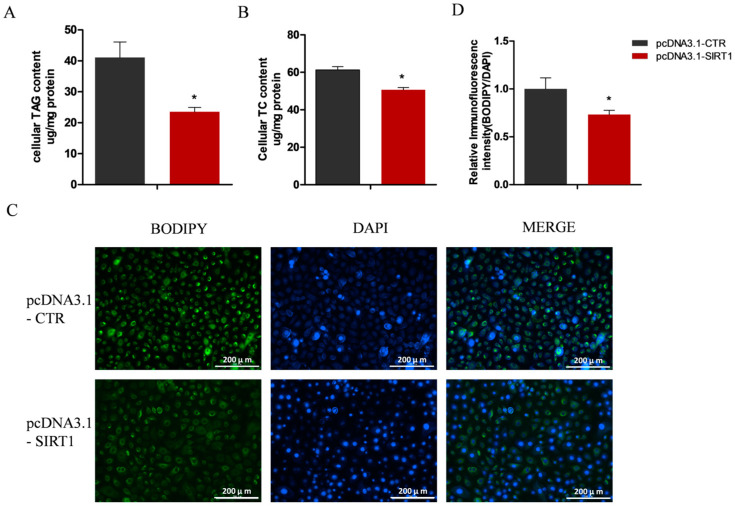
SIRT1 overexpression in GMECs regulates lipid metabolism. (**A**) SIRT1 overexpression significantly decreased the content of triacylglycerol (TAG) in GMECs. (**B**) SIRT1 overexpression reduced the cellular cholesterol levels. (**C**) Overexpression of SIRT1 inhibited the accumulation of lipid droplets. Cellular nuclei were stained with a blue signal, while the lipid droplets were labeled with a green signal. Scale bar = 200 μm. (**D**) The relative fluorescence intensity of the lipid droplets. Data shown are mean ± SEM. * *p* < 0.05.

Preliminary data indicated that changes in the SIRT1 expression led to the variation in the lipid accumulation in the GMECs. To determine whether the alterations in SIRT1 expression affect the intracellular contents of TAG and lipid droplets, we extracted the total TAG and cholesterol from the GMECs. Consistent with the observed transcriptional changes, the knockdown of *SIRT1* led to a significant increase in the cellular contents of TAG and cholesterol in the GMECs (*p* < 0.05; Figure 4A,B). Furthermore, the abundance of lipid droplets, which are the cellular organelles for storing neutral lipids, was also substantially increased upon the *SIRT1* knockdown (*p* < 0.05; Figure 4C,D). Conversely, *SIRT1* overexpression led to a significant decrease in the intracellular accumulation of TAG (*p* < 0.05; Figure 5A), cholesterol (*p* < 0.05; Figure 5B), and lipid droplets (*p* < 0.05; Figure 5C,D). These findings confirm the crucial role of SIRT1 in negatively regulating lipid synthesis in GMECs.

### 2.5. SIRT1 Enhanced ATGL Promoter Activity via FOXO1 Binding Sites

**Figure 6:**
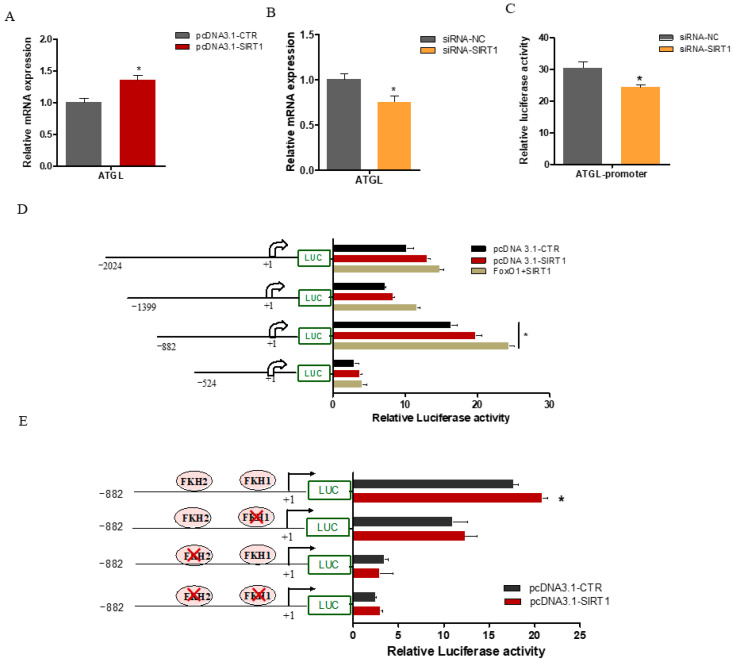
SIRT1 promotes ATGL transcription activity through FOXO1. (**A**,**B**) The expression of ATGL after SIRT1 overexpression or inhibition. (**C**) SIRT1 knockdown significantly decreased the ATGL promoter activity. (**D**) Effects of SIRT1 and FOXO1 on ATGL promoter activity. GMECs were co-transfected with different lengths of the ATGL promoter and plasmid expressing SIRT1 or empty vector for 48 h. Cells were harvested and luciferase activity assay was performed to detect the effect of SIRT1 on the ATGL promoter activity. In addition, to detect the effect of FOXO1 on the ATGL promoter activity, cells were co-transfected with different lengths of the ATGL promoter and plasmid expressing SIRT1 and FOXO1 for 48 h. (**E**) SIRT1 increases ATGL promoter activity in a FOXO1-dependent manner. GMECs were co-transfected with wild-type ATGL promoter (−882 + 216 bp) or constructs containing individually or simultaneously mutated FOXO1 binding sites and pcDNA3.1-SIRT1 for 48 h. Data are presented as mean ± SEM; *, *p* < 0.05. Note: SIRT1, Sirtuin 1; FOXO1, Forkhead box protein O1; ATGL, Adipose Triglyceride Lipase; FKH, FOXO1 binding sites; Red cross, Mutation site.

A previous study has shown that the knockdown of *SIRT1* decreases lipolysis in adipocytes by modulating the transcriptional activity of *ATGL* [16]. To further explore the molecular mechanisms underlying the regulation of *ATGL* by SIRT1 in GMECs, cells were transfected with pcDNA3.1-SIRT1 for 48 h. We found that the overexpression or knockdown of SIRT1 significantly upregulated (*p* < 0.05; Figure 6A) or reduced (*p* < 0.05; Figure 6B) the mRNA expression levels of *ATGL*, respectively. Furthermore, *SIRT1* knockdown significantly decreased the *ATGL* promoter activity (*p* < 0.05; Figure 6C). To further investigate the effect of *SIRT1* on the *ATGL* promoter activity, cells were co-transfected with pcDNA3.1-SIRT1 and an *ATGL* construct promoter for 48 h. Our results demonstrated that SIRT1 overexpression enhanced the *ATGL* promoter activity in a dose-dependent manner (Figure 6D). Interestingly, the co-overexpression of *SIRT1* and *FOXO1* led to a more pronounced increase in the *ATGL* promoter activity, particularly within the −882 to +216 bp region (*p* < 0.05; Figure 6D). Our recent study has shown that *FOXO1* promotes *ATGL* transcription through FOXO1 binding sites (FKH) located in the *ATGL* promoter [15]. To further elucidate the role of these *FOXO1* binding sites in mediating the stimulatory effect of SIRT1 on *ATGL* transcription, we performed site-directed mutagenesis assays. We found that *SIRT1* overexpression increased the *ATGL* promoter activity in groups with single FKH1 mutations compared to the empty vector group (Figure 6E). However, the ability of SIRT1 to enhance the *ATGL* promoter activity was nearly abolished when both the FKH1 and FKH2 sites were mutated (Figure 6E). These findings suggest that SIRT1 regulates lipid metabolism in GMECs by modulating the transcriptional activity of *ATGL*, an effect mediated at least in part by the interaction of SIRT1 with the transcription factor FOXO1 on the ATGL promoter.

## 3. Discussion

Goat milk is rich in nutrients, particularly milk fat, which offers numerous health benefits to humans [17,18]. Previous studies have shown that the medium-chain and short-chain fatty acids in goat milk can effectively dissolve excess cholesterol, increase the secretion of gastrointestinal hormones, and improve the intestinal flora, thereby promoting the absorption and digestion of nutrients in the human body [19,20]. Understanding the regulatory mechanisms of fatty acid synthesis is of paramount scientific importance for manipulating the milk composition. In this study, we found that the *SIRT1* expression was significantly elevated during peak lactation compared to the dry period. Using the knockdown and overexpression approaches, we observed that reducing the *SIRT1* levels promotes fatty acid synthesis and the cellular TAG content. In addition, *SIRT1* modulates the *ATGL* promoter activity through the transcriptional regulation of *FOXO1*, suggesting that *SIRT1* may play an important role in regulating lipid metabolism in GMECs.

Studies on SIRT1 have primarily focused on diseases such as obesity; however, SIRT1 has also been found to regulate lipid metabolism gene expression through a series of signaling pathways [21,22]. A previous study has shown that *SIRT1* can suppress the expression of *PPARG*, thereby reducing lipid synthesis in adipose tissue [8]. The SIRT1/AMPK pathway controls fatty acid production by downregulating the expression of *SREBP1* (a key regulator of lipogenesis) [23]. Furthermore, *SIRT1* also reduces the expression of SREBP2, thereby controlling cholesterol production and increasing the intracellular lipid storage [24,25]. These results are consistent with our findings in GMECs, where the overexpression of SIRT1 reduced the expression levels of *SREBP1* and *PPARG*. Notably, *SREBP1* and *PPARG* are key transcriptional regulators that induce the expressions of functional genes involved in the lactation process [26,27]. In addition, *SIRT1* upregulates the expressions of serine/threonine protein kinases, which, in turn, suppresses the expressions of *ACC* and *FASN*, thereby reducing the fat deposition in the liver [28,29]. In the present study, SIRT1 knockdown significantly increased the expressions of *SREBP1* and *FASN,* suggesting that de novo fatty acid synthesis is impeded in GMECs, resulting in decreased intracellular levels of short- and medium-chain fatty acids. Moreover, our previous findings indicate that *PPARG* regulates the expression of the *SCD1* gene by binding to the promoter of the *SCD1* gene [30]. Therefore, we hypothesize that the effect of SIRT1 on the changes in *SCD* expression is likely mediated through *PPARG* in GMECs.

Previous studies have shown that the activation of hypothalamic *SIRT1* gene expression can significantly reduce the serum triglyceride levels caused by acute alcohol exposure and reduce the accumulation of lipid droplets in the livers of mice [31]. SIRT1 activator treatment has also been shown to improve serum lipid profiles by lowering the total cholesterol, low-density lipoprotein (LDL) cholesterol, and triglyceride concentrations [32]. Furthermore, in a diabetic mouse model, elevated SIRT1 protein levels in liver, muscle, and fat cells were found to enhance the high-density lipoprotein (HDL) cholesterol function and prevent the development of diabetic kidney disease [22]. In the present study, SIRT1 knockdown or overexpression increased or reduced the intracellular TAG and cholesterol contents in the GMECs, respectively, consistent with previous findings. Therefore, SIRT1 plays a critical role in facilitating neutral lipid synthesis.

The overexpression or inhibition of SIRT1 alters the expression patterns of genes associated with lipid metabolism. *LIPIN*, a phosphatidate phosphatase enzyme, catalyzes a key step in the synthesis of triglycerides [33]. Knockout *SIRT1* in mouse liver increases the expression of *LIPIN1*, leading to lipid accumulation in the liver. Conversely, in AML-12 hepatocytes, the overexpression of *SIRT1* prevents lipid accumulation and reduces the expression of *LIPIN1* [34]. In addition, *PPARG* directly binds to the *LIPIN1* promoter, thereby promoting its expression and increasing the cellular content of TAGs in buffalo mammary epithelial cells [35]. In this study, *SIRT1* overexpression significantly decreased the *LIPIN1* mRNA levels, suggesting that SIRT1 may directly control *LIPIN1* transcription and indirectly suppress *LIPIN1* expression by inhibiting the transcription factor *PPARG*. Moreover, *DGAT1* and *DGAT2* are key enzymes responsible for TAG synthesis [36]. Expression analyses of AGPAT6 variants have shown a strong positive correlation between increased *AGPAT6* expression and a high milk fat percentage [37,38]. In this study, the knockdown of SIRT1 increased the expressions of *DGAT1*, *DGAT2*, and *AGPAT6*, which could explain the increased TAG levels in the GMECs.

Milk fat is an indicator of the nutritional value of milk, with 98% of its composition consisting of triglycerides (TAGs), the majority of which are stored in lipid droplets [39]. Our finding showed that the overexpression of *SIRT*1 reduced the lipid droplet accumulation in the GMECs. Knockout of the *SIRT1* gene in mice increased the liver lipid droplet content and reduced the TAG catabolism [40], whereas the activation of *SIRT1* by resveratrol significantly suppressed the lipid droplet accumulation in mouse liver [41], supporting our results in GMECs. Additionally, the PLIN2 protein plays a critical role in the formation, stabilization, and regulation of intracellular lipid droplets [42]. *PLIN2* knockout decreased the liver lipid droplet accumulation and TAG levels [43]. The overexpression of SIRT1 reduced the expression of *PLIN2*, which is consistent with the decreased lipid droplet accumulation in GMECs. Furthermore, the overexpression of *ELOVL6* resulted in an accumulation of TAGs in GMECs. In our study, the expression of *ELOVL6* did not change after SIRT1 activation, suggesting that this specific regulatory mechanism requires further in-depth research.

ATGL is a key enzyme that catalyzes lipolysis and serves as a central regulator of systemic energy homeostasis [44]. In 3T3-L1 adipocytes, *SIRT1* knockout reduced the ATGL expression, leading to a decrease in TAG lipolysis [16]. We observed similar results in the GMECs utilized in this study. Previous studies have shown that the overexpression of *SIRT1* alone does not affect *ATGL* promoter activity; however, co-transfection with *FOXO1* significantly increases *ATGL* promoter activity, which is consistent with our findings that SIRT1 overexpression enhances *ATGL* promoter activity. This discrepancy may be attributed to differences in species and physiological conditions. Additionally, our previous study demonstrated that *FOXO1* regulates *ATGL* transcription by binding to the FKH binding sites of the *ATGL* promoter, with a notably higher affinity for the FKH2 site [15]. Furthermore, SIRT1 activates *FOXO1* through its deacetylase activity, allowing *FOXO1* to regulate various target genes [45]. In this study, we found that the simultaneous mutation of both the FKH1 and FKH2 sites produced similar results to the FKH2 single mutation, nearly abolishing SIRT1’s ability to enhance *ATGL* promoter activity. This indicates that the FKH2 site plays a predominant role in mediating the regulatory effects of *SIRT1* on *ATGL* transcription. These findings suggest that the interaction between *SIRT1* and *FOXO1* at specific binding regions of the *ATGL* promoter is crucial for the SIRT1-mediated upregulation of ATGL transcription.

## 4. Materials and Methods

### 4.1. Animals and Tissue Collection

In the present study, we selected 5 healthy non-lactating Saanen dairy goats (from the experimental farm at Northwest A&F University) and collected samples of rumen, liver, kidney, mammary gland, small intestine, heart, spleen, lung, muscle, and sebum. The goat mammary gland tissues at early lactation, peak lactation, mid-lactation, and during the dry period were obtained through surgical methods. All the tissue samples were immediately washed with DEPC water and stored in liquid nitrogen for RNA extraction.

### 4.2. Cell Culture

Five healthy peak-lactation Xinong Saanen dairy goats were used to isolate GMECs, following a previously described method [46]. The protocols for cell purification and authentication have been detailed in earlier studies [47,48]. Briefly, mammary glands were cut into 1 mm^3^ cubes and placed in cell dishes. These tissue explants were then incubated at 37 °C with 5% CO_2_ until the cells migrated out from the tissue blocks. The detached cells were then maintained in a growth medium containing DMEM/F12 basal medium (11320-033, Invitrogen Corporation, Waltham, MA, USA) supplemented with 5 μg/mL bovine insulin (16634, Sigma, St. Louis, MO, USA), hydrocortisone, epidermal growth factor (PHG0311, Invitrogen), 100 U/mL penicillin/streptomycin (080092569, Harbin Pharmaceutical Group, Harbin, China), and 10% fetal bovine serum (10099-141, Invitrogen, USA). Prior to further experimentation, the GMECs were incubated for 48 h in lactation medium supplemented with prolactin (L6520, 2.5 μg/mL, Sigma, USA) to induce lactogenesis.

### 4.3. Overexpression Vector Construction

To generate a SIRT1 overexpression construct, the full-length coding sequence of goat SIRT1 was cloned using gene-specific primers designed with *BamHI* and *XhoI* restriction enzyme recognition sites. Then, the SIRT1 fragment was subsequently cloned into vector pcDNA3.1, which had been pre-digested with BamHI and XhoI(R0136, R0146, NEB, Ipswich, MA, USA). The FOXO1 overexpression vector pcDNA3.1- FOXO1 was stored in our laboratory. The primers were as follows: sense: 5′-CG**GGATCC***GCCACC*ATGGCGGACGAGGCGGCGCTCG-3′; antisense: 5′- CCG**CTCGAG**TTATGATTTGTTTGATGGATAGT-3′. Note: Bold indicates restriction sites, and eukaryotic Kozak sequences are represented in italic font.

### 4.4. RNA Interference Experiment

Goat-specific SIRT1 small interfering RNA (siRNA) was designed based on the sequence of SIRT1 from the NCBI and synthesized by the Tsingke company (Beijing, China). The sequences were as follows: sense: 5′-CCAGUAGCACUAAUUCCAATT-3′; antisense: 5′-UUGGAAUUAGUGCUACUGGTT-3′. The siRNA was then transfected into the GMECs at a final concentration of 50 nM using RNAiMAX(13778150, Invitrogen, USA), according to the manufacturer’s instructions.

### 4.5. RNA Extraction and Real-Time Quantitative PCR (RT-qPCR)

GMECs were seeded in 12-well plates and then transfected with a SIRT1 overexpression vector or siRNA for 48 h. Total RNA was extracted from the GMECs and collected tissues using Trizol reagent (Invitrogen Corp., Carlsbad, CA, USA) following the manufacturer’s instructions. The RNA concentration and quantity were determined using a spectrophotometer (Nanodrop 2000, Thermo, USA). An amount of 1 ug total RNA was used to synthesize cDNA by utilizing the PrimeScript RT Reagent Kit (RR047A, Takara, Otsu, Japan). RT-qPCR was performed on a Real-Time PCR Detection System, and the condition followed previous studies [21,30]. Relative expression levels of the target genes were calculated using the 2^−ΔΔCt^ method and were normalized to ubiquitously expressed transcript (UXT) and ribosomal protein S9 (RPS9). The primer sequences for the target genes are listed in Table 1.

### 4.6. Western Blot

GMECs were seeded in 6-well plates and then transfected with a SIRT1 overexpression vector or siRNA for 48 h. Then, cells were lysed in RIPA lysis buffer (R0010, Solarbio, Beijing, China) contained with cOmplete Protease Inhibitor Cocktail (04693132001, Roche Diagnostics Ltd., Mannheim, Germany). The protein concentration was determined using a BCA protein assay kit (23227, Thermo Fisher Scientific, Rockford, IL, USA), and 20 μg total protein was separated on a 10% SDS-PAGE gel. Then, the protein was transferred onto a PVDF membrane and blocked with BSA in TBST. SIRT1 polyclonal antibody (13161-1-AP, Proteintech, Wuhan, China, 1:1000) and β-actin mouse monoclonal antibody (CW0096, CW Biotech, Beijing, China, 1:1000) were used as primary antibodies, incubated overnight at 4 °C. Goat anti-rabbit (CW0103, CW Biotech, Beijing, China, 1:2000) and goat anti-mouse (CW0102, CW Biotech, Beijing, China,1:2000) HRP-conjugated IgG were used as secondary antibodies. ImageJ was used to analyze the protein expression levels.

### 4.7. Cellular TAG and Cholesterol Analysis

GMECs were cultured in six-well plates and transfected with either SIRT1-pc DNA3.1 or siSIRT1 for 48 h, and then cells were harvested for cellular TAG and cholesterol detection. Then, the TAG and cholesterol in the cells were extracted by the TAG (E1013, Applypen Technologies, Beijing, China) and cholesterol (E1015, Applygen Technologies, Beijing, China) assay kits. The sample contents were read at 550 nm using a Biotek Microplate Reader (Winooski, VT, USA). The relative content of triacylglycerol was corrected by intracellular protein levels and displayed as micrograms per milligram of protein (μg/mg protein), for which the protein content was determined via a BCA Protein Assay Kit (23227, Thermo Fisher Scientific, Rockford, IL, USA).

### 4.8. BODIPY Staining

GMECs were seeded into 12-well plates and transfected with either SIRT1-pc DNA3.1 or siSIRT1 for 48 h. Then, GMECs were fixed with 4% paraformaldehyde for 30 min after being washed with PBS 3 times. The lipid droplets were stained with 0.1% BODIPY 493/503 (D3922, Invitrogen), and cell nuclei were counterstained using DAPI solution (C1006, Beyotime, Shanghai, China). Following the staining procedures, the cells were rinsed three times with PBS. The lipid droplet images were captured using a Cell Imaging Reader (BioTek Instruments Inc., Winooski, VT, USA). The fluorescence intensity of BODIPY was used to indicate the content of lipid droplets, which was normalized by DAPI staining.

### 4.9. Cell Transfection and Luciferase Assay

ATGL promoter luciferase reporter plasmids were stored in our laboratory. To explore the effect of SIRT1 on the ATGL promoter activity, cells were co-transfected with SIRT1-pcDNA3.1 and ATGL promoter. To explore the effect of FOXO1 on the ATGL promoter activity, cells were co-transfected with SIRT1-pcDNA3.1, FOXO1-pcDNA3.1, and ATGL promoter for 48 h. Briefly, GMECs were seeded in 48-well plates, and then the 300 ng ATGL promoter and overexpression plasmid were transfected into the cells when they reached 80%–90% confluence. A Renilla luciferase vector was used as an internal control and was co-transfected with the ATGL promoter construct at a 1:50 ratio into the cells. After transfection for 48 h, the cells were lysed and luciferase activity was detected using the Dual-Luciferase Reporter Assay System (E1910, Promega, Madison, WI, USA).

### 4.10. Statistical Analysis

The data presented in this study are expressed as mean ± SEM, and all experiments were conducted in triplicate. Prior to statistical analysis, the Jarque–Bera method was used to assess the normality of the data distributions. The statistical analysis was performed using SPSS 20.0. Statistical analysis was performed with Student’s *t*-test for only two groups, and one-way ANOVA was performed with Duncan’s test for multiple comparisons. The significance level was set at * *p* < 0.05 and ** *p* < 0.01.

## 5. Conclusions

In conclusion, our data indicate that SIRT1 suppresses the synthesis of fatty acids, triglycerides, and lipid droplets by modulating the gene expressions involved in lipid metabolism in GMECs. In addition, SIRT1 regulates the transcription of ATGL by modulating FOXO1 binding to the ATGL promoter. These findings highlight the critical importance of the SIRT1-FOXO1 regulatory axis in controlling ATGL-mediated lipolysis, and they provide important mechanistic insights into the transcriptional regulation of lipid metabolism in mammary epithelial cells.

## Figures and Tables

**Table 1:** Real-time quantitative PCR primers.

Gene		Primer Sequence (5′-3′)	Size (bp)
*UXT*	F R	CAGCTGGCCAAATACCTTCAA GTGTCTGGGACCACTGTGTCAA	125
*RPS9*	F R	CCTCGACCAAGAGCTGAAG CCTCCAGACCTCACGTTTGTTC	64
*FASN*	F R	GGGCTCCACCACCGTGTTCCA GCTCTGCTGGGCCTGCAGCTG	226
*SCD1*	F R	CCATCGCCTGTGGAGTCAC GTCGGATAAATCTAGCGTAGCA	257
*ELOVL6*	F R	GGAAGCCTTTAGTGCTCTGGTC ATTGTATCTCCTAGTTCGGGTGC	205
*XDH*	F R	GATCATCCACTTTTCTGCCAATG CCTCGTCTTGGTGCTTCCAA	100
*PLIN2*	F R	GATGAGACCACGGCAGATG GTCAACTATTTCCCGCACAAG	120
*DGAT1*	F R	CCACTGGGACCTGAGGTGTC GCATCACCACACACCAATTCA	111
*GPAM*	F R	ATTGACCCTTGGCACGATAG AACAGCACCTTCCCACAAAG	188
*AGPAT6*	F R	AAGCAAGTTGCCCATCCTCA AAACTGTGGCTCCAATTTCGA	101
*SREBP1*	F R	ACGCCATCGAGAAACGCTAC GTGCGCAGACTCAGGTTCTC	181
*PPARG*	F R	CCTTCACCACCGTTGACTTCT GTATCAGGCTCCACTTTGATTGC	145
*LIPIN1*	F R	GAGGGGAAGAAACACCACAA GTAGCTGACGCTGGACAACA	202
*XBP1*	F R	AGTTAAGACAGCGGTTGGGG CACTCCATTCCCCTTGGTCT	71

## Data Availability

The data supporting the findings of this study are available within the article. For any further inquiries, contact the corresponding author.
